# D-dimer-albumin ratio (DAR) as a new biomarker for predicting preoperative deep vein thrombosis after geriatric hip fracture patients

**DOI:** 10.1186/s13018-023-04139-z

**Published:** 2023-08-31

**Authors:** Wei Yao, Kaihua Zhang, Qiaomei Lv, Ziyang Deng, Wenbo Ding

**Affiliations:** 1 Department of Orthopedics, Dandong Central Hospital, China Medical University, Dandong, China; 2 Department of Orthopedics, Third Affiliated Hospital of Jinzhou Medical University, Jinzhou, China; 3 Department of Oncology, Dandong Central Hospital, China Medical University, Dandong, China

**Keywords:** Hip fracture, Geriatric, Preoperative deep vein thrombosis, Prognosis, D-dimer-albumin ratio

## Abstract

**Purpose:**

Hip fractures in the elderly are complicated by preoperative deep vein thrombosis (DVT). The objective of this study is to determine the usefulness of blood-based biomarkers, particularly the D-dimer-albumin ratio (DAR), in predicting preoperative DVT.

**Methods:**

A retrospective observational study was carried out on 1149 patients from a single hospital, and subsequently validated on an additional 626 patients from a separate hospital. The aim was to evaluate the prognostic and predictive value of 10 biomarkers, with a specific emphasis on DAR, in both cohorts. The primary measure of interest was the occurrence of preoperative DVT.

**Results:**

The ratio of D-dimer to albumin demonstrated superior predictive capability for preoperative DVT in older patients with hip fractures compared to other biomarkers (AUC = 0.677). Using the optimal cutoff point of 0.24, high DAR was significantly associated with preoperative DVT (OR 3.45, 95% CI 2.00–5.95). Notably, all the DAR definitions detailed above were successfully validated in an external, independent cohort.

**Conclusions:**

DAR may be a valuable biomarker for predicting preoperative DVT in elderly patients with hip fractures.

**Supplementary Information:**

The online version contains supplementary material available at 10.1186/s13018-023-04139-z.

## Introduction

Hip fracture is a prevalent condition among elderly patients, with significant morbidity and mortality [1–4]. Within the first year of injury, approximately 30% of elderly patients with hip fractures will succumb [5], while survivors are faced with an increasing disease burden that detrimentally affects their quality of life [6]. The main factors contributing to hip fractures in the elderly population are osteoporosis, muscle weakness, and low-impact trauma [7, 8]. Given the accelerated aging of the global population, the incidence of hip fractures is steadily rising and projected to reach 4.5 million cases by 2050, posing a considerable challenge to the public health sector [9, 10]. Surgery currently serves as an effective intervention for treating hip fractures. Depending on the fracture pattern, recommended surgical approaches for intertrochanteric and subtrochanteric fractures include intramedullary nailing or sliding/dynamic hip screws (DHS) [11, 12]. For femoral neck fractures, total hip arthroplasty (THA) or hemiarthroplasty is recommended [13]. In most cases, surgery significantly improves patients' pain, functional abilities, and overall quality of life [11, 14, 15]. Nevertheless, perioperative complications often compromise the benefits of surgery for many elderly patients, leading to increased disease burden and mortality [16–18].

Preoperative deep vein thrombosis (DVT) is a common complication among elderly patients with hip fractures, which significantly affects their prognosis [18–20]. The asymptomatic nature of most deep vein thrombi often renders clinical diagnoses unreliable, resulting in undetected cases of DVT [21, 22]. Furthermore, detached embolisms from the blood vessel wall can lead to serious and potentially fatal risks, such as pulmonary embolism [23]. With the rising incidence of hip fractures in the elderly population [4], the prompt and accurate diagnosis and timely intervention for DVT present significant clinical challenges [24]. Reliable and evidence-based predictive tools, including biomarkers, are essential for clinical doctors to make informed decisions soon after admission and prevent adverse outcomes [25].

D-dimer serves as a vital component of the diagnostic algorithm for DVT and is widely used to predict and exclude DVT [26, 27]. Plasma D-dimer is formed through the degradation of fibrin filaments by fibrinolytic enzymes during the coagulation process [28]. Consequently, increased D-dimer levels reflect the occurrence of thrombogenesis and lysis within the body, serving as a non-invasive marker of thrombus formation [29]. Despite its high sensitivity, the clinical utility of D-dimer in older populations is limited due to its relatively low specificity [30] and tendency to increase with age [31]. Low albumin levels also play a significant role in the development of DVT. Albumin inhibits fibrin polymerization and platelet aggregation while possessing heparin-like properties [32]. Moreover, decreased albumin levels can lead to reduced plasma colloid osmotic pressure, increased blood viscosity, and ultimately thrombus formation [33]. Several previous studies have emphasized the importance of serum albumin levels in predicting the prognosis and complications in orthopedic patients before surgery. Specifically, elderly patients with hip fractures who have lower serum albumin levels are more susceptible to perioperative complications, and preoperative DVT formation is independently associated with low albumin levels [20, 34, 35].

Although the aforementioned biomarkers have been widely utilized in clinical practice [36, 37], whether the combination of biomarkers can enhance the prognostic ability for preoperative DVT in patients with hip fractures remains uncertain. No studies have been conducted to our knowledge thus far to evaluate the combined application of D-dimer and albumin for the prediction of preoperative DVT events in elderly patients with hip fractures. Addressing these gaps necessitates a comprehensive evaluation of various biomarker combinations, particularly focusing on the newly developed combined DAR as an efficient biomarker for predicting preoperative DVT in the elderly with hip fractures. Further investigation requires larger sample sizes from multiple hospitals to facilitate a more detailed and accurate analysis.

## Materials and methods

### Study design and data sources

To compose the training set, we conducted a retrospective analysis of elderly hip fracture patients admitted to our institution between February 2015 and February 2023. We also collected data from an independent institution to facilitate external validation, analyzing records of elderly hip fracture patients from January 2018 to January 2023. The institutional review boards of both hospitals granted ethical approval for the study and waived the need for informed consent.

### Patient selection

The inclusion criterion verifies patients' eligibility in the study if they are diagnosed with hip fractures in old age and whose diagnosis is based on imaging (X-ray, CT, or MRI), physical examination, or intraoperative assessment by an orthopedic surgeon. Participants are excluded if they meet any of the following criteria: (1) < 60 years of age; (2) multiple, pathologic, or open fractures; (3) lack of perioperative ultrasound examination after admission; (4) didn't undergo laboratory tests such as hematology within 24 h of admission; (5) prior utilization of anticoagulant drugs or diagnosis of peripheral vascular disease.

### Variables

The demographic data comprise information on age, gender, smoking, and alcohol consumption. The study encompasses data on past medical history, including hypertension, diabetes, chronic obstructive pulmonary disease (COPD), cardiovascular disease, stroke, chronic liver disease, deep vein puncture, venous thromboembolism, and cancer. Additionally, the study encompasses data on the type of fracture (femoral neck, intertrochanteric, or subtrochanteric), injury-to-admission time, and duration of bedridden time.

### Laboratory indicators

Blood samples were collected from hip fracture patients within 24 h of admission, and relevant biomarkers were collected. The collected biomarkers include D-dimer (ug/L), albumin (g/L), neutrophils (× 10^9^/L), lymphocytes (× 10^9^/L), red blood cells (× 10^12^/L), white blood cells (× 10^9^/L), blood glucose (mmol/L), platelets (× 10^9^/L), mean platelet volume (FL), red cell distribution width (%), alanine aminotransferase (IU/L), aspartate aminotransferase (IU/L), cholesterol (mmol/L), low-density lipoprotein (mmol/L), high-density lipoprotein (mmol/L), triglycerides (mmol/L), fibrinogen (g/L), activated partial thrombin time (s), prothrombin time (s), and thrombin time (s).

### Definition of outcome

The principal outcome is the incidence rate of preoperative DVT. Color Doppler ultrasonography stands out as the most reliable diagnostic tool for examining lower limb vessels [38]. Our institution necessitates all hip fracture patients to undergo routine ultrasound examinations within 24 h of admission and every 3–5 days thereafter while awaiting surgery. The efficacy of this approach lies in its ability to carefully examine all deep veins from the inguinal ligament to the ankle. Moreover, only seasoned ultrasound physicians evaluate these images, and their diagnoses are based on predetermined imaging features. The specific criteria [39] used to diagnose DVT are typically characterized by the presence of abnormal echoes, venous stenosis with lumen obstruction or incompressibility, the absence of a blood flow signal in the occluded segment, and reduced blood flow in the distal end in comparison with the proximal end of the occluded vein. Upon admission, hip fracture patients in our establishment must elevate their limbs to improve blood flow and discourage thrombotic events. For those diagnosed with preoperative DVT, our management strategy complies with the recommendations provided by the American College of Chest Physicians [40]. Such an approach typically involves administering low-molecular-weight heparin such as enoxaparin sodium at a dosage of 4000 IU, one dose per day after 24 h of admission. On the other hand, in the absence of DVT, patients are advised to engage in regular limb movement, proper hydration, and the use of intermittent pneumatic compression devices if necessary.

### Statistical analysis

Continuous variables are presented as median (± IQR), while categorical variables are noted as absolute numbers (%). A bilateral *P* value < 0.05 was deemed statistically significant. Any missing values in continuous variables were imputed using a median-based approach. In this study, we examined and assessed four blood-based biomarkers in hip fracture patients. Two of the biomarkers, namely D-dimer and neutrophils, showed a significant increase in adverse outcomes, while the remaining two biomarkers, lymphocytes, and albumin, demonstrated a significant decrease in adverse outcomes. We used these four biomarkers in combination to determine the optimal predictability of preoperative DVT in hip fracture patients. We identified a D-dimer to albumin ratio that exhibited the highest accuracy for predicting the occurrence of preoperative DVT.

We assessed the predictive performance of various biomarkers to determine their utility in predicting the occurrence of preoperative deep vein thrombosis (DVT) in hip fracture patients. To this end, we carried out a receiver operating characteristic (ROC) curve analysis and calculated the area under the ROC curve (AUC), a metric that measures the accuracy of predictions. The optimal cutoff point was determined using the Youden index, a measure that maximizes the difference between the true positive rate and the false positive rate, thereby creating a balance between sensitivity and specificity.

To determine the impact of various covariates on the occurrence of preoperative deep vein thrombosis (DVT) in hip fracture patients, we utilized multivariate logistic regression. The analysis generated odds ratios (ORs) and corresponding 95% confidence intervals (CIs). In the initial phase, univariate regression was performed, and any variable with a *p* value < 0.10 was selected for the multivariate logistic regression analysis. For our analysis, we carefully selected the covariates for the logistic regression model, considering previous research [18, 41] and clinical expertise. To enhance the reliability of our findings, we conducted a sensitivity analysis using propensity score matching [42]. We employed the *E*-value approach to evaluate the robustness of the observed association with unmeasured confounding [43, 44]. We used an online calculator (https://www.evalue-calculator.com/evalue/) to calculate the *E*-value, which provides an estimate of the minimum strength of association needed for an unmeasured confounder to account for the observed association. Additionally, we partitioned patients into four groups based on the quartile distribution of the DAR values, specifically *Q*1 (< 0.05), *Q*2 (0.05–0.10), *Q*3 (0.10–0.19), and *Q*4 (> 0.19). This categorical approach allowed us to determine the dose–response relationship more accurately between the biomarkers and preoperative DVT. All statistical analyses were performed using R software version 4.0.3.

## Results

A training set consisting of 1149 elderly hip fracture patients was incorporated into the analysis. Among them, 141 cases (12.3%) were indicated to have preoperative deep vein thrombosis (DVT). Additionally, the validation set of 626 elderly hip fracture patients was included, out of which 81 cases (12.9%) were diagnosed with preoperative DVT for further examination (see Additional file 1: Fig. S1). The optimal cutoff value for DAR was established to be 0.24. The baseline characteristics of geriatric hip fracture patients from both the training and validation sets are shown in Table 1, stratified based on the optimal cutoff value for DAR. Remarkably, patients with elevated levels of DAR in both cohorts displayed a prolonged interval between injury and admission, which may have preemptively boosted the onset of VTE complications. Additionally, they showed a distinct tendency to have a more pronounced prevalence of VTE-related diseases such as hypertension and chronic obstructive pulmonary disease documented in their medical history.

**Table 1:** Baseline characteristics stratified by baseline DAR levels

Variables	Training set			External validation set		
	DAR < 0.24^&^ (*n* = 940)	DAR ≥ 0.24^&^ (*n* = 209)	*p* value	DAR < 0.24^&^ (*n* = 545)	DAR ≥ 0.24^&^ (*n* = 81)	*p* value
Demographic						
Age, × years	76.00 (66.00–82.25)	77.00 (67.00–83.00)	0.13	76.00 (67.00–83.00)	76.00 (67.00–83.00)	0.88
Female gender	584 (62.13)	121 (57.89)	0.26	313 (57.43)	49 (60.49)	0.60
Smoking	153 (16.28)	48 (22.97)	0.02	85 (15.60)	16 (19.75)	0.34
Alcohol	95 (10.11)	31 (14.83)	0.05	61 (11.19)	10 (12.35)	0.76
Comorbidities						
Hypertension	464 (49.36)	130 (62.20)	< 0.001	270 (49.54)	52 (64.20)	0.01
Diabetes	215 (22.87)	53 (25.36)	0.44	129 (23.67)	22 (27.16)	0.49
COPD	96 (10.21)	42 (20.10)	< 0.001	56 (10.28)	15 (18.52)	0.03
Cardiovascular disease	286 (30.43)	70 (33.49)	0.39	168 (30.83)	29 (35.80)	0.37
Stroke	248 (26.38)	56 (26.79)	0.90	148 (27.16)	26 (32.10)	0.35
Chronic liver disease	38 (4.04)	15 (7.18)	0.05	20 (3.67)	6 (7.41)	0.12
History of deep venipuncture	11 (1.17)	5 (2.39)	0.17	9 (1.65)	3 (3.70)	0.21
History of deep VTE	77 (8.19)	27 (12.92)	0.03	52 (9.54)	13 (16.05)	0.07
Neoplasms	85 (9.04)	18 (8.61)	0.84	56 (10.28)	4 (4.94)	0.13
Operation						
Fracture type						
Femoral neck fracture	505 (53.72)	99 (47.37)	0.39	251 (46.06)	35 (43.21)	0.62
Intertrochanteric fracture	377 (40.11)	102 (48.80)		270 (49.54)	42 (51.58)	
Subtrochanteric fracture	58 (6.17)	8 (3.83)		24 (4.40)	4 (4.94)	
Admission time						
< 6 h	471 (50.11)	144 (68.90)	< 0.001	275 (50.46)	56 (69.14)	0.001
6–12 h	148 (15.74)	27 (12.92)		92 (16.88)	11 (13.58)	
> 12 h	321 (34.15)	38 (18.18)		178 (32.66)	14 (17.28)	
Bedridden time	5.00 (3.00–7.00)	5.00 (4.00–8.00)	0.01	5.00 (4.00–7.00)	5.00 (4.00–8.00)	0.12
Biology						
D-dimer, × mg/L	2.92 (1.49–4.85)	11.69 (10.00–19.50)	< 0.001	2.79 (1.20–4.63)	10.66 (10.00–20.00)	< 0.001
ALB, × g/dL	38.00 (35.00–41.00)	37.00 (35.00–40.00)	< 0.001	38.00 (35.00–41.00)	36.00 (33.00–39.00)	< 0.001
NEU, × 10^9/L	6.40 (4.90–8.10)	7.00 (5.50–9.00)	< 0.001	6.30 (4.80–8.10)	7.10 (5.60–9.00)	0.01
LYM count, × 10^9/L	1.20 (0.90–1.60)	1.10 (0.80–1.50)	0.003	1.20 (0.90–1.60)	1.00 (0.70–1.30)	0.001

Data are presented as median (interquartile range) or n (%); DAR: D-dimer—albumin ratio

^&^the best cutoff for the DAR was 0.24, which was identified by Youden’s index

*COPD* chronic obstructive pulmonary disease, *VTE* venous thromboembolism, *ALB* albumin, *NEU* neutrophils, *LYM* lymphocyte

**Fig. 1:**
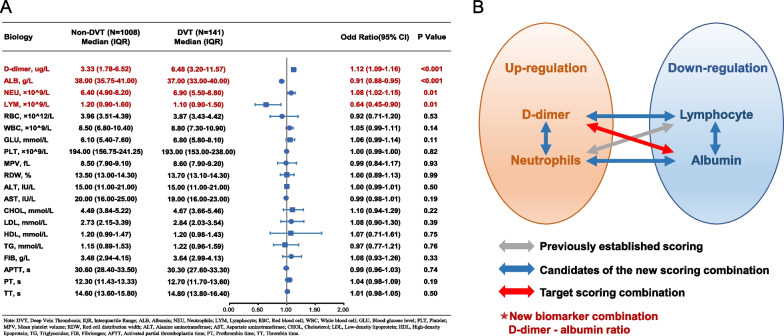
**A** Baseline data and univariate analysis for each laboratory factors. **B** Schematic chart for the combination of laboratory factors in this study

**Fig. 2:**
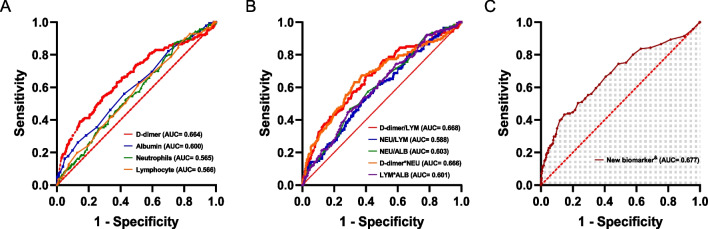
ROC curves analysis to evaluate the predictive value of each combination for Preoperative DVT in patients with hip fractures from the training set: The DAR **C** showed the highest accuracy for the prediction of Preoperative DVT compared with established scores including other laboratory factors **A**, **B** in hip fracture patients. ROC, receiver operating characteristic; AUC, area under the curve. &New biomarker combination: D-dimer-albumin ratio

**Fig. 3:**
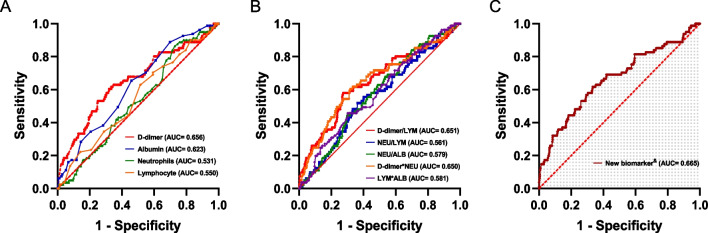
ROC curves analysis to evaluate the predictive value of each combination for Preoperative DVT in patients with hip fractures from the validation set: The DAR (**C**) showed the highest accuracy for the prediction of Preoperative DVT compared with established scores including other laboratory factors (**A**, **B**) in hip fracture patients. ROC, receiver operating characteristic; AUC, area under the curve. &New biomarker combination: D-dimer-albumin ratio

The collected biomarkers, along with their respective baseline levels, are depicted in Fig. 1A. After thorough analysis, four blood biomarkers, and six prospective combinations were identified and subsequently evaluated, as displayed in Fig. 1B. Among these combinations, the accuracy of DAR in predicting preoperative DVT in elderly hip fracture patients was the highest compared to single biomarkers and their combinations (Fig. 2A, B), with an AUC of 0.677 (Fig. 2C). DAR demonstrated significantly greater diagnostic accuracy compared to alternative biomarker combinations (*p* < 0.001, see Table 2). Notably, DAR maintained its high level of accuracy in predicting preoperative DVT within the external validation set with an AUC of 0.665 (Fig. 3), and its predictive ability was far stronger than other biomarkers (*p* = 0.04, see Table 3). For further elucidation, a detailed account of the predictive ability of each biomarker in the trial group and validation group can be found in the appendix (see Additional file 1: Figs. S2 and S3).

**Table 2:** Assessment of the characteristic parameters of each biomarker in the training set

Variables	AUC (95% CI)	ACC (%, 95% CI)	SEN (%, 95% CI)	SPE (%, 95% CI)	PPV (%, 95% CI)	NPV (%, 95% CI)	DeLong test (*p* value)
DAR	0.677 (0.626–0.729)	80.2 (80.1–80.2)	43.3 (35.1–51.4)	85.3 (83.1–87.5)	29.2 (23.0–35.4)	91.5 (89.7–93.3)	Reference^&^
D-dimer	0.664 (0.612–0.715)	69.4 (69.3–69.4)	53.9 (45.7–62.1)	71.5 (68.7–74.3)	20.9 (16.8–25.1)	91.7 (89.8–93.7)	< 0.001
ALB	0.600 (0.549–0.651)	57.4 (57.4–57.5)	56.0 (47.8–64.2)	57.6 (54.6–60.7)	15.6 (12.4–18.8)	90.4 (88.1–92.6)	–
NEU	0.565 (0.516–0.613)	33.3 (33.3–33.4)	87.2 (81.7–92.7)	25.8 (23.1–28.5)	14.1 (11.8–16.4)	93.5 (90.6–96.4)	–
LYM	0.566 (0.516–0.615)	50.7 (50.6–50.7)	61.7 (53.7–69.7)	49.1 (46.0–52.2)	14.5 (11.7–17.3)	90.2 (87.7–92.7)	–

*ALB* albumin, *NEU* Neutrophils, *LYM* lymphocyte, *CI* confidence interval, *AUC* the area under the curve, *ACC* accuracy, *SEN* sensitivity, SPE specificity, PPV positive predictive value, NPV negative predictive value

^&^A comparison of AUC was performed using the DeLong test

**Table 3:** Assessment of the characteristic parameters of each biomarker in the validation set

Variables	AUC (95% CI)	ACC (%, 95% CI)	SEN (%, 95% CI)	SPE (%, 95% CI)	PPV (%, 95% CI)	NPV (%, 95% CI)	DeLong test (*p* value)
DAR	0.665 (0.596–0.734)	66.1 (66.1–66.2)	61.7 (51.1–72.3)	66.8 (62.8–70.7)	21.6 (16.3–27.0)	92.2 (89.5–94.8)	Reference^&^
D-dimer	0.656 (0.587–0.725)	67.1 (67.0–67.2)	60.5 (49.8–71.1)	68.1 (64.2–72.0)	22.0 (16.5–27.4)	92.1 (89.4–94.7)	0.04
ALB	0.623 (0.561–0.685)	55.6 (55.5–55.7)	65.4 (55.1–75.8)	54.1 (49.9–58.3)	17.5 (13.2–21.8)	91.3 (88.3–94.4)	–
NEU	0.531 (0.469–0.594)	34.8 (34.8–34.9)	86.4 (79.0–93.9)	27.2 (23.4–30.9)	15.0 (11.8–18.2)	93.1 (89.1–97.0)	–
LYM	0.550 (0.483–0.616)	50.5 (50.4–50.6)	63.0 (52.4–73.5)	48.6 (44.4–52.8)	15.4 (11.5–19.3)	89.8 (86.4–93.3)	–

*ALB* albumin, *NEU* neutrophils, *LYM* lymphocyte, *CI* confidence interval, *AUC* the area under the curve, *ACC* accuracy, *SEN* sensitivity, *SPE* specificity, *PPV* positive predictive value, *NPV* negative predictive value

^&^A comparison of AUC was performed using the DeLong test

In order to examine the relationship between DAR and preoperative DVT in elderly hip fracture patients, we conducted a detailed evaluation, and the comprehensive outcomes are presented in Table 4. Notably, in univariate regression analysis, elevated levels of DAR were identified as significantly associated with preoperative DVT (OR 4.43, 95% CI 3.04–6.45). This association remained significant after controlling for potential confounding factors such as age, smoking, hypertension, diabetes, COPD, stroke, history of deep venous catheterization, history of venous thromboembolism, and bedridden time (OR 4.02, 95%CI 2.62–6.18). The consistency of the results was well established in the results of the external validation set (OR 4.15, 95% CI 2.21–7.79, as shown in Table 5). The detailed results of the multivariate regression analysis can be found in the appendix (see Additional file 1: Tables S1 and S3). The *e*-value, signifying the minimum threshold required to nullify the association between DAR and preoperative DVT, was determined to be 7.50, with a lower limit of 4.68.

**Table 4:** Unadjusted and adjusted associations between preoperative DVT and DAR based on different cutoff values in the training set

DAR	Events, *n* (%)	Unadjusted OR (95% CI)	*P*	Multivariable regression adjusted OR (95% CI)	*P*	PSM adjusted OR (95% CI)	*P*
Continuous							
Per SD	NA	1.84 (1.59–2.12)	< 0.001	1.78 (1.52–2.10)	< 0.001	NA	NA
Best cutoff^&^							
< 0.24	80 (8.5)	1 [Reference]	< 0.001	1 [Reference]	< 0.001	1 [Reference]	< 0.001
≥ 0.24	61 (29.2)	4.43 (3.04–6.45)		4.02 (2.62–6.18)		3.45 (2.00–5.95)	
Quartile							
Q1 (< 0.05)	19 (7.1)	1 [Reference]	< 0.001*	1 [Reference]	< 0.001*	1 [Reference]	< 0.001*
Q2 (0.05–0.10)	25 (7.2)	1.02 (0.55–1.89)		0.64 (0.32–1.26)		0.59 (0.30–1.15)	
Q3 (0.10–0.19)	32 (12.5)	1.85 (1.02–3.35)		1.42 (0.75–2.69)		1.27 (0.73–2.19)	
Q4 (> 0.19)	65 (23.1)	3.91 (2.27–6.73)		2.84 (1.57–5.16)		2.61 (1.63–4.19)	

*SD* standard deviation, *NA* not available, *OR* odds ratio, *PSM* propensity scores matching

*p for trend; ^&^the best cutoff for the DAR was 0.24, which was identified by Youden’s index

**Table 5:** Unadjusted and adjusted associations between preoperative DVT and DAR based on different cutoff values in the validation set

DAR	Events, n (%)	Unadjusted OR (95% CI)	*P*	Multivariable regression adjusted OR (95% CI)	*P*	PSM adjusted OR (95% CI)	*P*
Continuous							
Per SD	NA	1.79 (1.47–2.18)	< 0.001	1.83 (1.45–2.30)	< 0.001	NA	NA
Best cutoff^&^							
< 0.24	41 (8.5)	1 [Reference]	< 0.001	1 [Reference]	< 0.001	1 [Reference]	0.01
≥ 0.24	33 (22.8)	4.21 (2.45–7.25)		4.15 (2.21–7.79)		3.02 (1.37–6.65)	
Quartile							
Q1 (< 0.05)	12 (7.7)	1 [Reference]	< 0.001*	1 [Reference]	< 0.001*	1 [Reference]	< 0.001*
Q2 (0.05–0.10)	16 (9.8)	1.05 (0.47–2.34)		0.72 (0.30–1.71)		0.90 (0.41–1.95)	
Q3 (0.10–0.19)	11 (7.5)	1.99 (0.98–4.06)		1.68 (0.78–3.60)		1.18 (0.58–2.37)	
Q4 (> 0.19)	35 (21.9)	4.47 (2.29–8.75)		3.90 (1.88–8.07)		3.51 (1.99–6.18)	

*SD* standard deviation, *NA* not available, *OR* odds ratio, *PSM* propensity scores matching

*p for trend; ^&^ the best cutoff for the DAR was 0.24, which was identified by Youden’s index

**Fig. 4:**
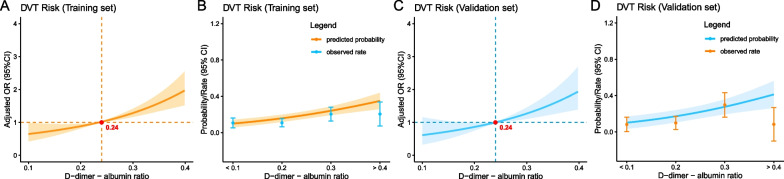
Propensity score matching adjusted odds ratio (OR) for preoperative DVT according to levels of DAR in the training (**A**) and validation set (**C**) on a continuous scale. Predicted probabilities and the observed rate of preoperative DVT: Training set (**B**); Validation set (**D**)

Upon conducting an evaluation of DAR as a continuous variable, the adjusted OR for preoperative DVT was identified to be 1.78 (95% CI 1.52–2.10) with each 1-SD rise in DAR (see Table 4). Furthermore, upon analyzing DAR as quartiles, clear evidence of a dose–response relationship was found between DAR and preoperative DVT not only in the training set but in the validation set as well (p for trend < 0.001). These findings, which are consolidated in Tables 4 and 5, with a graphical representation in Fig. 4, show that increased levels of DAR are logically linked to a higher incidence of preoperative DVT.

We also conducted propensity score matching as a sensitivity analysis, thus minimizing the potential influence of confounding factors. Upon matching, all variables between the experimental and validation groups displayed a balanced distribution, indicating that any difference between the groups could not be attributed to confounding factors (see Additional file 1: Tables S2 and S4). Excitingly, the newly analyzed propensity-matched association between DAR and preoperative DVT remained significant in both the experimental group (OR 3.45, 95% CI 2.00–5.95, see Table 4) and the validation group (OR 3.02, 95% CI 1.37–6.65, see Table 5), reinforcing the accuracy of our findings.

## Discussion

In this retrospective cohort study, we evaluated various combinations of biomarkers to determine their capacity for predicting preoperative deep vein thrombosis (DVT) among elderly hip fracture patients. Remarkably, our findings highlight a significant statistical association between elevated levels of DAR when its values exceed the designated threshold of 0.24, and an increased susceptibility to preoperative DVT. In fact, among all the biomarker combinations tested, DAR demonstrated the highest predictive ability for this condition. Importantly, our study findings were scrutinized and verified across a broad range of patients in an independent large cohort, underscoring the generalizability and reliability of our observations.

A plethora of studies have extensively investigated the association between D-dimer and albumin with preoperative deep vein thrombosis (DVT). The elevation of D-dimer concentration during admission may arise from stress and inflammatory responses [45]. Typically, augmented D-dimer levels signify escalated activation of coagulation and fibrinolysis systems, with the latter being an anticoagulation mechanism that maintains vascular permeability, blood flow, and tissue repair [46]. Once thrombosis formation occurs, plasminogen activation triggers the fibrinolytic process, which breaks down the fibrin clot into fibrin products. Of all these products, solely the D-D cross-linking site can reflect lytic activity after thrombosis formation [47]. Consequently, clinical D-dimer measurement serves as a sensitive indicator for screening and diagnosing new thrombus formations [48]. Nonetheless, while D-dimer exhibits high sensitivity, its specificity is low [27], and D-dimer levels increase with age, potentially leading to more false positive results in elderly patients [26]. Prior research has employed various combinations of age and D-dimer as critical thresholds, thereby significantly enhancing predictive accuracy for DVT [31]. Nonetheless, evaluating the efficacy of joint diagnostic tests that account for both age and D-dimer levels remains controversial across different medical contexts [49].

Hypoalbuminemia is typically viewed as a manifestation of malnutrition. Inflammation augments capillary permeability, leading to the expansion of the interstitial space and amplification of the distribution volume of albumin, resulting in hypoalbuminemia due to the inflammatory state [32]. A compelling case has been reported linking hypoalbuminemia to elevated fibrinogen levels and platelet aggregation [50]. Since albumin is solely produced in the liver, low serum albumin levels serve as a marker of poor liver reserve and hence a likely deficiency in anticoagulation factors, thereby increasing the risk of DVT [51]. Clinical studies of patients with cirrhosis have strongly associated hypoalbuminemia with increased DVT occurrence [52]. Similarly, hypoalbuminemia can be caused by renal loss, and studies have indicated that low serum albumin significantly predicts DVT occurrence in nephrotic syndrome [53]. Thus, low levels of albumin can also serve as a marker of a hypercoagulable state [54].

Although DAR is a novel combination in this study, it is established that D-dimer and albumin alone can serve as predictive factors for preoperative deep vein thrombosis (DVT) in hip fracture patients. To illustrate, Zhao et al. conducted a retrospective study on 1515 elderly hip fracture patients and reported that low albumin levels (< 35 g/L) (OR = 1.52, *p* = 0.04) and D-dimer levels > 1.59 mg/L (OR = 2.19, *p* < 0.001) were independently correlated with preoperative DVT [20]. Zhang et al. developed an age-adjusted D-dimer index for preoperative DVT screening in 2759 elderly hip fracture patients [31]. Further, Chang et al. documented the predictive significance of preoperative D-dimer levels for venous thrombosis among lower limb fracture patients [45]. Additionally, from the analysis of 930 elderly hip fracture patients, Tian et al. reported that preoperative low albumin levels were significantly linked with poor prognosis and heightened risk of hospital readmissions [55]. Interestingly, Panteli et al. unveiled that hip fracture patients with hypoalbuminemia during admission significantly experienced an increased mortality risk [35]. Nevertheless, the association between DAR and preoperative DVT is intricate and possibly confounded by various factors. Consequently, we incorporated the *E*-value in this study to assess the potential impact of unmeasured confounders on our conclusions. The *E*-value represents the minimum strength of association between unmeasured confounders and both the exposure and outcome variables needed to fully account for the observed association, while also controlling for measured confounders [44]. In this study, we calculated the *E*-value to be 7.50, with a lower limit of 4.68. These values indicate that unmeasured confounders are insufficient to completely negate the observed association between DAR and preoperative deep venous thrombosis, thus underscoring the robustness of our findings. Additionally, more research is warranted to thoroughly investigate this potential association and determine the optimal strategy for employing DAR as a predictive marker for preoperative DVT in hip fracture patients.

D-dimer and albumin are the most prevalent biomarkers employed in clinical practice, providing a low-cost, accessible modality for identifying high-risk patients. Furthermore, both are modifiable risk factors, allowing physicians to take proactive measures. Currently, researchers are exploring enhanced methods to manage preoperative DVT in hip fracture patients, underscoring the significance of preoperative screening, perioperative care, and correction of nutritional status as effective strategies for minimizing preoperative DVT risk [56, 57]. It has also been suggested that the introduction of geriatric orthopedic surgeons in orthopedic wards treating elderly hip fracture patients may improve perioperative complications and poor prognosis [58]. Ultimately, obtaining accurate prognoses and identifying preoperative DVT can greatly enhance communication between clinicians and patients, especially when family members are involved.

The strength of this study lies in its vast patient population, encompassing 1146 patients in the training set and 624 patients in the validation set. Additionally, all biomarkers obtained in the training set were also acquired in the validation set, thus limiting the risk of selection bias. However, we must acknowledge several limitations of our study. Firstly, retrospective studies may be subject to various biases and potential unmeasured confounding factors, and missing data. Therefore, a prospective trial is required to validate our findings. Secondly, our analysis only considered biomarkers routinely used in clinical practice, thus disregarding other potentially more intricate combinations with better predictive abilities than DAR. Thirdly, D-dimer and albumin levels may change during hospitalization, and while we attempted to reduce confounding effects by using solely baseline levels of D-dimer and albumin drawn upon admission, these changes were not analyzed across the hospitalization period. Finally, our study only investigated whether DAR correlates with preoperative DVT in hip fracture patients during hospitalization, other studies are required to evaluate whether DAR has an impact on postoperative DVT and long-term survival rates in this patient group.

## Conclusions

This extensive study has successfully identified DAR as a straightforward, synergistic, and highly accessible biomarker, effectively predicting the likelihood of preoperative DVT in elderly patients with hip fractures. The admission DAR value can be utilized to identify and categorize high-risk preoperative DVT patients. Following this intriguing discovery, future clinical trials are warranted to assess the utility of this biomarker in hip fracture management and ascertain its efficacy in routine clinical practice.

### Supplementary Information

**Additional file 1.** Additional materials about this study (including 1. Study flow chart; 2. ROC curves for the training and validation sets; 3. Multivariate analysis and propensity score matching results for preoperative DVT in the training and validation sets).

## Data Availability

All the data used and analyzed during the current study are available from the corresponding author upon reasonable request.

## References

[1] Maffulli N, Aicale R (2022). Proximal femoral fractures in the elderly: a few things to know, and some to forget. Medicina (Kaunas).

[2] Aletto C, Aicale R, Pezzuti G, Bruno F, Maffulli N (2020). Impact of an orthogeriatrician on length of stay of elderly patient with hip fracture. Osteoporos Int.

[3] Zhang C, Feng J, Wang S, Gao P, Xu L, Zhu J, Jia J, Liu L, Liu G, Wang J, Zhan S, Song C (2020). Incidence of and trends in hip fracture among adults in urban China: a nationwide retrospective cohort study. PLoS Med.

[4] Bhandari M, Swiontkowski M (2017). Management of acute hip fracture. N Engl J Med.

[5] Abrahamsen B, van Staa T, Ariely R, Olson M, Cooper C (2009). Excess mortality following hip fracture: a systematic epidemiological review. Osteoporos Int.

[6] Griffin XL, Parsons N, Achten J, Fernandez M, Costa ML (2015). Recovery of health-related quality of life in a United Kingdom hip fracture population. The Warwick Hip Trauma Evaluation—a prospective cohort study. Bone Joint J..

[7] Chen YP, Kuo YJ, Hung SW, Wen TW, Chien PC, Chiang MH, Maffulli N, Lin CY (2021). Loss of skeletal muscle mass can be predicted by sarcopenia and reflects poor functional recovery at one year after surgery for geriatric hip fractures. Injury.

[8] Kanis JA, Odén A, McCloskey EV, Johansson H, Wahl DA, Cooper C (2012). A systematic review of hip fracture incidence and probability of fracture worldwide. Osteoporos Int.

[9] Veronese N, Maggi S (2018). Epidemiology and social costs of hip fracture. Injury.

[10] Gullberg B, Johnell O, Kanis JA (1997). World-wide projections for hip fracture. Osteoporos Int.

[11] Marsillo E, Pintore A, Asparago G, Oliva F, Maffulli N (2022). Cephalomedullary nailing for reverse oblique intertrochanteric fractures 31A3 (AO/OTA). Orthop Rev.

[12] Gargano G, Poeta N, Oliva F, Migliorini F, Maffulli N (2021). Zimmer Natural Nail and ELOS nails in pertrochanteric fractures. J Orthop Surg Res.

[13] Coomber R, Porteous M, Hubble MJW, Parker MJ (2016). Total hip replacement for hip fracture: surgical techniques and concepts. Injury.

[14] Handoll HH, Sherrington C, Mak JC (2011). Interventions for improving mobility after hip fracture surgery in adults. Cochrane Database Syst Rev.

[15] LeBlanc KE, Muncie HL, LeBlanc LL (2014). Hip fracture: diagnosis, treatment, and secondary prevention. Am Fam Physician.

[16] Knauf T, Hack J, Barthel J, Eschbach D, Schoeneberg C, Ruchholtz S, Buecking B, Aigner R (2020). Medical and economic consequences of perioperative complications in older hip fracture patients. Arch Osteoporos.

[17] Chen Y, Liang S, Wu H, Deng S, Wang F, Lunzhu C, Li J (2022). Postoperative delirium in geriatric patients with hip fractures. Front Aging Neurosci.

[18] Wang T, Guo J, Long Y, Yin Y, Hou Z (2022). Risk factors for preoperative deep venous thrombosis in hip fracture patients: a meta-analysis. J Orthop Traumatol.

[19] Sathiyakumar V, Greenberg SE, Jahangir AA, Mir HH, Obremskey WT, Sethi MK (2015). Impact of type of surgery on deep venous thrombi and pulmonary emboli: a look at twenty seven thousand hip fracture patients. Int Orthop.

[20] Zhao K, Wang Z, Tian S, Hou Z, Chen W, Zhang Y (2022). Incidence of and risk factors for pre-operative deep venous thrombosis in geriatric intertrochanteric fracture patients. Int Orthop.

[21] Thorson CM, Ryan ML, Van Haren RM, Curia E, Barrera JM, Guarch GA, Busko AM, Namias N, Livingstone AS, Proctor KG (2012). Venous thromboembolism after trauma: a never event?*. Crit Care Med.

[22] Lobastov K, Sautina E, Alencheva E, Bargandzhiya A, Schastlivtsev I, Barinov V, Laberko L, Rodoman G, Boyarintsev V (2021). Intermittent pneumatic compression in addition to standard prophylaxis of postoperative venous thromboembolism in extremely high-risk patients (IPC SUPER): a randomized controlled trial. Ann Surg.

[23] Wenger N, Sebastian T, Engelberger RP, Kucher N, Spirk D (2021). Pulmonary embolism and deep vein thrombosis: similar but different. Thromb Res.

[24] Righini M, Le Gal G, Bounameaux H (2015). Venous thromboembolism diagnosis: unresolved issues. Thromb Haemost.

[25] Biomarkers and surrogate endpoints: preferred definitions and conceptual framework (2001). Clinical pharmacology and therapeutics 69:89-95. 10.1067/mcp.2001.113989 10.1067/mcp.2001.113989 11240971

[26] Olson JD (2015). D-dimer: an overview of hemostasis and fibrinolysis, assays, and clinical applications. Adv Clin Chem.

[27] Aguilar C, del Villar V (2007). Combined D-dimer and clinical probability are useful for exclusion of recurrent deep venous thrombosis. Am J Hematol.

[28] Weitz JI, Fredenburgh JC, Eikelboom JW (2017). A test in context: D-dimer. J Am Coll Cardiol.

[29] Soomro AY, Guerchicoff A, Nichols DJ, Suleman J, Dangas GD (2016). The current role and future prospects of D-dimer biomarker. Eur Heart J Cardiovasc Pharmacother.

[30] Chopard R, Albertsen IE, Piazza G (2020). Diagnosis and treatment of lower extremity venous thromboembolism: a review. JAMA.

[31] Zhang K, Zhu Y, Tian Y, Tian M, Li X, Zhang Y (2021). Role of a new age-adjusted D-dimer cutoff value for preoperative deep venous thrombosis exclusion in elderly patients with hip fractures. J Orthop Surg Res.

[32] Chi G, Gibson CM, Liu Y, Hernandez AF, Hull RD, Cohen AT, Harrington RA, Goldhaber SZ (2019). Inverse relationship of serum albumin to the risk of venous thromboembolism among acutely ill hospitalized patients: analysis from the APEX trial. Am J Hematol.

[33] Violi F, Ceccarelli G, Loffredo L, Alessandri F, Cipollone F, D'Ardes D, D'Ettorre G, Pignatelli P, Venditti M, Mastroianni CM, Pugliese F (2021). Albumin supplementation dampens hypercoagulability in COVID-19: a preliminary report. Thromb Haemost.

[34] Ding K, Wang H, Jia Y, Zhao Y, Yang W, Chen W, Zhu Y (2022). Incidence and risk factors associated with preoperative deep venous thrombosis in the young and middle-aged patients after hip fracture. J Orthop Surg Res.

[35] Panteli M, Giannoudi MP, Lodge CJ, West RM, Pountos I, Giannoudis PV (2021). Mortality and medical complications of subtrochanteric fracture fixation. J Clin Med.

[36] Tan Z, Hu H, Wang Z, Wang Y, Zhang Y (2021). Prevalence and risk factors of preoperative deep venous thrombosis in closed patella fracture: a prospective cohort study. J Orthop Surg Res.

[37] Li Y, Jiang Q, Zhou X, Wu M, Chen J, Liu H, Dai S, Zheng Z, Zhao X, Zhang C, Shi Z, Zhang H, Gu J, Huang Z, Yin G, Zhao S (2022). A prospective marker for the prediction of postoperative deep venous thrombosis: Neutrophil extracellular traps. Front Cell Dev Biol.

[38] Kruger PC, Eikelboom JW, Douketis JD, Hankey GJ (2019). Deep vein thrombosis: update on diagnosis and management. Med J Aust.

[39] Barrosse-Antle ME, Patel KH, Kramer JA, Baston CM (2021). Point-of-care ultrasound for bedside diagnosis of lower extremity DVT. Chest.

[40] Falck-Ytter Y, Francis CW, Johanson NA, Curley C, Dahl OE, Schulman S, Ortel TL, Pauker SG, Colwell CW (2012). Prevention of VTE in orthopedic surgery patients: antithrombotic Therapy and Prevention of Thrombosis, 9th ed: American College of Chest Physicians Evidence-Based Clinical Practice Guidelines. Chest.

[41] Zhang BF, Wei X, Huang H, Wang PF, Liu P, Qu SW, Li JH, Wang H, Cong YX, Zhuang Y, Zhang K (2018). Deep vein thrombosis in bilateral lower extremities after hip fracture: a retrospective study of 463 patients. Clin Interv Aging.

[42] Stukel TA, Fisher ES, Wennberg DE, Alter DA, Gottlieb DJ, Vermeulen MJ (2007). Analysis of observational studies in the presence of treatment selection bias: effects of invasive cardiac management on AMI survival using propensity score and instrumental variable methods. JAMA.

[43] Mathur MB, Ding P, Riddell CA, VanderWeele TJ (2018). Web site and R package for computing E-values. Epidemiology.

[44] VanderWeele TJ, Ding P (2017). Sensitivity analysis in observational research: introducing the E-value. Ann Intern Med.

[45] Chang W, Wang B, Li Q, Zhang Y, Xie W (2021). Study on the risk factors of preoperative deep vein thrombosis (DVT) in patients with lower extremity fracture. Clin Appl Thromb Hemost.

[46] Cheng J, Fu Z, Zhu J, Zhou L, Song W (2020) The predictive value of plasminogen activator inhibitor-1, fibrinogen, and D-dimer for deep venous thrombosis following surgery for traumatic lower limb fracture. Annals of palliative medicine 9:3385–3392. 10.21037/apm-20-1604 10.21037/apm-20-1604 32954761

[47] Cosmi B, Palareti G (2010). Update on the predictive value of D-dimer in patients with idiopathic venous thromboembolism. Thromb Res.

[48] Goodacre S, Sampson FC, Sutton AJ, Mason S, Morris F (2005). Variation in the diagnostic performance of D-dimer for suspected deep vein thrombosis. QJM Mon J Assoc Phys.

[49] Takach Lapner S, Julian JA, Linkins LA, Bates SM, Kearon C (2016). Questioning the use of an age-adjusted D-dimer threshold to exclude venous thromboembolism: analysis of individual patient data from two diagnostic studies. J Thromb Haemost JTH.

[50] Kim SB, Chi HS, Park JS, Hong CD, Yang WS (1999). Effect of increasing serum albumin on plasma D-dimer, von Willebrand factor, and platelet aggregation in CAPD patients. Am J Kidney Dis.

[51] Gulley D, Teal E, Suvannasankha A, Chalasani N, Liangpunsakul S (2008). Deep vein thrombosis and pulmonary embolism in cirrhosis patients. Dig Dis Sci.

[52] Northup PG, McMahon MM, Ruhl AP, Altschuler SE, Volk-Bednarz A, Caldwell SH, Berg CL. Coagulopathy does not fully protect hospitalized cirrhosis patients from peripheral venous thromboembolism. Am J Gastroenterol. 2006;101:1524–1528; quiz 1680. 10.1111/j.1572-0241.2006.00588.x 10.1111/j.1572-0241.2006.00588.x 16863556

[53] Ambler B, Irvine S, Selvarajah V, Isles C (2008). Nephrotic syndrome presenting as deep vein thrombosis or pulmonary embolism. Emerg Med J EMJ.

[54] Folsom AR, Lutsey PL, Heckbert SR, Cushman M (2010). Serum albumin and risk of venous thromboembolism. Thromb Haemost.

[55] Tian M, Wang Z, Zhu Y, Tian Y, Zhang K, Li X (2023). Incidence, causes, and risk factors of unplanned readmissions in elderly patients undergoing hip fracture surgery: an observational study. Clin Interv Aging.

[56] Luksameearunothai K, Sa-Ngasoongsong P, Kulachote N, Thamyongkit S, Fuangfa P, Chanplakorn P, Woratanarat P, Suphachatwong C (2017). Usefulness of clinical predictors for preoperative screening of deep vein thrombosis in hip fractures. BMC Musculoskelet Disord.

[57] Wakabayashi H, Hasegawa M, Niimi R, Sudo A (2015). Clinical analysis of preoperative deep vein thrombosis risk factors in patients undergoing total hip arthroplasty. Thromb Res.

[58] Quaranta M, Miranda L, Oliva F, Migliorini F, Pezzuti G, Maffulli N (2021). Haemoglobin and transfusions in elderly patients with hip fractures: the effect of a dedicated orthogeriatrician. J Orthop Surg Res.

