# Supplementary material for: D-dimer-albumin ratio (DAR) as a new biomarker for predicting preoperative deep vein thrombosis after geriatric hip fracture patients
Source: J Orthop Surg Res. 2023 Aug 31;18:645. doi: 10.1186/s13018-023-04139-z (PMC10470167; doi:10.1186/s13018-023-04139-z)
Supplement: Supplementary file 1 — Additional file 1. Additional materials about this study (including 1. Study flow chart; 2. ROC curves for the training and validation sets; 3. Multivariate analysis and propensity score matching results for preoperative DVT in the training and validation sets). [file 13018_2023_4139_MOESM1_ESM.docx]

**Appendix:**

**eFigure1 Flow diagram of patients included in the cohort.**

**eFigure2 ROC curves of each marker for Preoperative DVT from the training set.**

**eFigure3 ROC curves of each marker for Preoperative DVT from the validation set.**

**eTable1 Multivariate Analysis for preoperative DVT in the training set.**

**eTable2 Patient Characteristics of the training set before and After Propensity Score Matching by the best cutoff value of the DAR.**

**eTable3 Multivariate Analysis for preoperative DVT in the validation set.**

**eTable4 Patient Characteristics of the validation set before and After Propensity Score Matching by the best cutoff value of the DAR.**


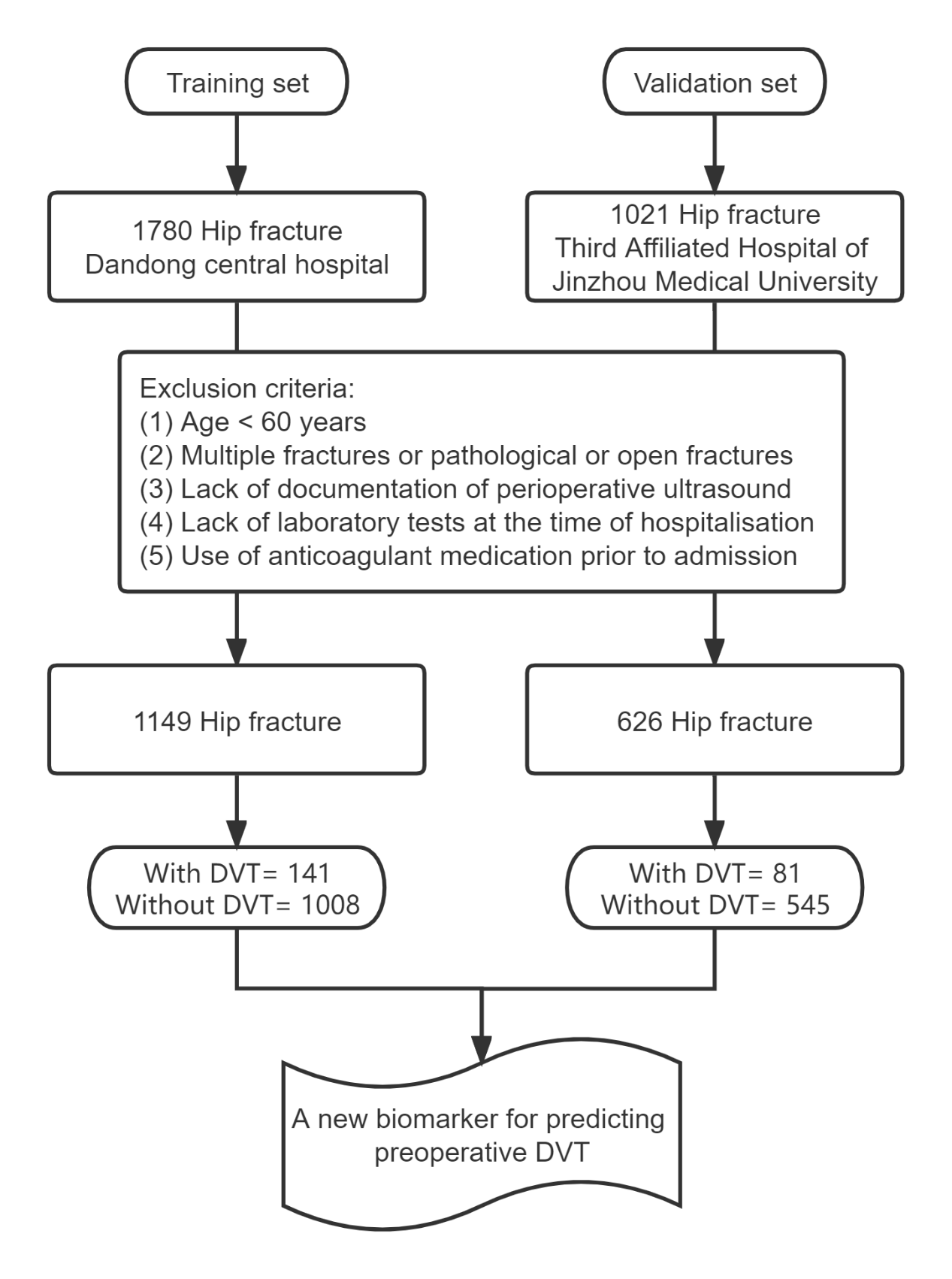
**eFigure1 Flow diagram of patients included in the cohort.**


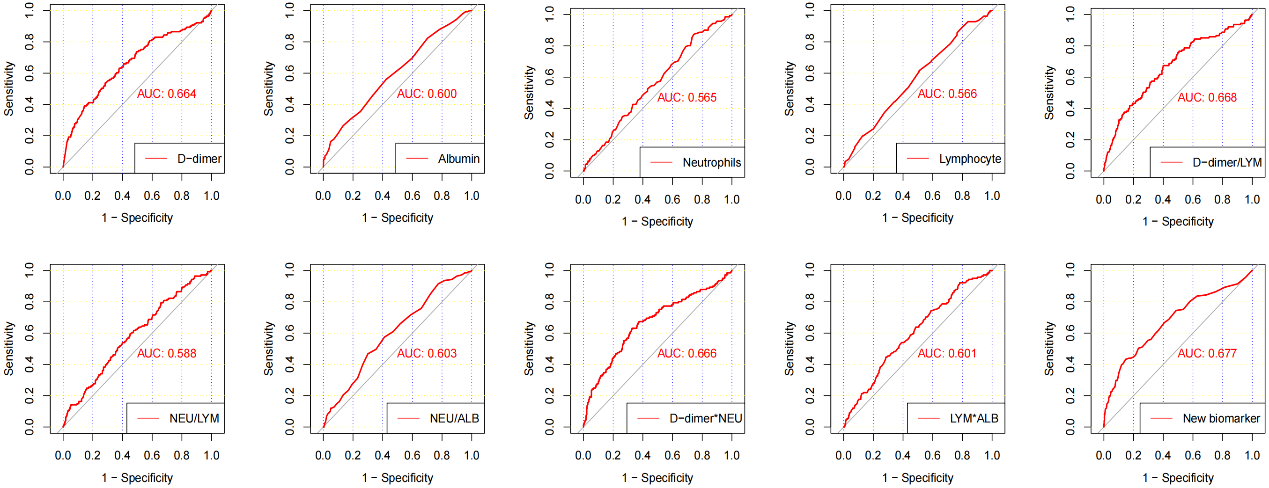


**eFigure2 ROC curves of each marker for Preoperative DVT from the training set.**


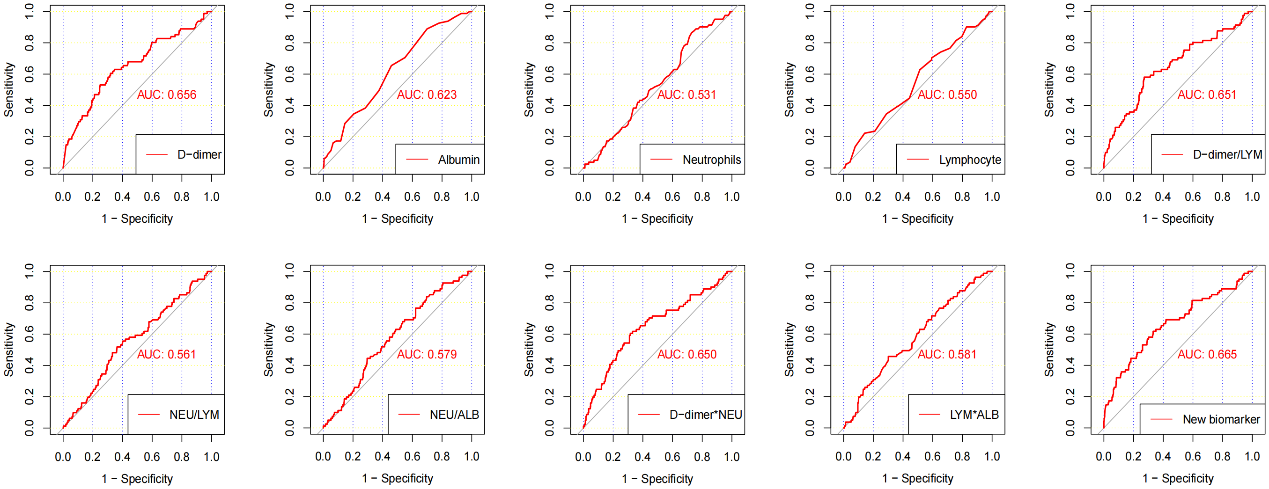


**eFigure3 ROC curves of each marker for Preoperative DVT from the validation set.eTable1 Multivariate Analysis for preoperative DVT in the training set**

| Characteristics | Univariate | | | Multivariate | | |
| --- | --- | --- | --- | --- | --- | --- |
|  | OR | 95%CI | P-value | OR | 95%CI | P-value |
| Demographic |  |  |  |  |  |  |
| Age, × years | 1.04 | 1.02-1.06 | <0.001 | 1.04 | 1.02-1.07 | <0.001 |
| Female gender | 1.05 | 0.73-1.51 | 0.78 | <NA> | <NA> | <NA> |
| Smoking | 2.46 | 1.66-3.65 | <0.001 | 2.90 | 1.78-4.75 | <0.001 |
| Alcohol | 1.51 | 0.91-2.50 | 0.11 | <NA> | <NA> | <NA> |
| Comorbidities |  |  |  |  |  |  |
| Hypertension | 2.03 | 1.40-2.95 | <0.001 | 1.49 | 0.96-2.30 | 0.08 |
| Diabetes | 1.99 | 1.37-2.90 | <0.001 | 1.71 | 1.11-2.65 | 0.02 |
| COPD | 1.71 | 1.06-2.74 | 0.03 | 0.82 | 0.46-1.46 | 0.50 |
| Cardiovascular disease | 1.01 | 0.69-1.48 | 0.95 | <NA> | <NA> | <NA> |
| Stroke | 1.46 | 1.00-2.13 | 0.05 | 0.63 | 0.39-1.03 | 0.07 |
| Chronic liver disease | 1.09 | 0.48-2.47 | 0.83 | <NA> | <NA> | <NA> |
| History of deep venipuncture | 9.75 | 3.57-26.62 | <0.001 | 3.91 | 1.14-13.40 | 0.03 |
| History of deep VTE | 8.34 | 5.37-12.96 | <0.001 | 7.55 | 4.39-13.02 | <0.001 |
| Neoplasms | 0.94 | 0.50-1.76 | 0.84 | <NA> | <NA> | <NA> |
| Operation |  |  |  |  |  |  |
| Fracture type | 0.92 | 0.69-1.22 | 0.55 | <NA> | <NA> | <NA> |
| Admission time | 1.17 | 0.96-1.42 | 0.12 | <NA> | <NA> | <NA> |
| Bedridden time | 1.11 | 1.06-1.15 | <0.001 | 1.09 | 1.05-1.14 | <0.001 |
| Laboratory findings |  |  |  |  |  |  |
| DAR^*^ | 4.43 | 3.04-6.45 | <0.001 | 4.02 | 2.62-6.18 | <0.001 |

*cutoff=0.24, which was identified by Youden’s index.

**eTable2 Patient Characteristics of the training set before and After Propensity Score Matching by the best cutoff value of DAR**

| Characteristics | Before matching | | | After matching | | |
| --- | --- | --- | --- | --- | --- | --- |
|  | DAR＜0.24  (n=940) | DAR ≥ 0.24  (n=209) | SMD | DAR＜0.24  (n=207) | DAR ≥ 0.24  (n=207) | SMD |
| Demographic |  |  |  |  |  |  |
| Age, × years | 76.00 (66.00-82.25) | 77.00 (67.00-83.00) | 0.12 | 77.00 (67.00-83.00) | 77.00 (67.00-83.00) | 0.05 |
| Female gender | 584 (62.13) | 121 (57.89) | 0.09 | 115 (55.83) | 121 (58.74) | 0.06 |
| Smoking | 153 (16.28) | 48 (22.97) | 0.17 | 44 (21.36) | 45 (21.84) | 0.01 |
| Alcohol | 95 (10.11) | 31 (14.83) | 0.14 | 27 (13.11) | 29 (14.08) | 0.03 |
| Comorbidities |  |  |  |  |  |  |
| Hypertension | 464 (49.36) | 130 (62.20) | 0.26 | 123 (59.71) | 128 (62.14) | 0.05 |
| Diabetes | 215 (22.87) | 53 (25.36) | 0.06 | 50 (24.27) | 51 (24.76) | 0.01 |
| COPD | 96 (10.21) | 42 (20.10) | 0.28 | 40 (19.42) | 39 (18.93) | 0.01 |
| Cardiovascular disease | 286 (30.43) | 70 (33.49) | 0.07 | 74 (35.92) | 69 (33.50) | 0.05 |
| Stroke | 248 (26.38) | 56 (26.79) | 0.01 | 50 (24.27) | 55 (26.70) | 0.06 |
| Chronic liver disease | 38 (4.04) | 15 (7.18) | 0.14 | 15 (7.28) | 15 (7.28) | <0.001 |
| History of deep venipuncture | 11 (1.17) | 5 (2.39) | 0.09 | 7 (3.40) | 5 (2.43) | 0.06 |
| History of deep VTE | 77 (8.19) | 27 (12.92) | 0.15 | 21 (10.19) | 26 (12.62) | 0.08 |
| Neoplasms | 85 (9.04) | 18 (8.61) | 0.02 | 22 (10.68) | 18 (8.74) | 0.07 |
| Operation |  |  |  |  |  |  |
| Fracture type |  |  | 0.07 |  |  | 0.05 |
| Femoral neck fracture | 505 (53.72) | 99 (47.37) |  | 95 (46.12) | 96 (46.60) |  |
| Intertrochanteric fracture | 377 (40.11) | 102 (48.80) |  | 98 (47.57) | 102 (49.51) |  |
| Subtrochanteric fracture | 58 (6.17) | 8 (3.83) |  | 13 (6.31) | 8 (3.88) |  |
| Admission time |  |  | 0.41 |  |  | 0.07 |
| < 6 h | 471 (50.11) | 144 (68.90) |  | 148 (71.84) | 141 (68.45) |  |
| 6h-12 h | 148 (15.74) | 27 (12.92) |  | 24 (11.65) | 27 (13.11) |  |
| >12 h | 321 (34.15) | 38 (18.18) |  | 34 (16.50) | 38 (18.45) |  |
| Bedridden time | 5.00 (3.00-7.00) | 5.00 (4.00-8.00) | 0.19 | 5.00 (4.00-7.00) | 5.00 (4.00-7.00) | 0.04 |

Data are presented as median (interquartile range) and n (%); DAR: D-dimer – albumin ratio; SMD: standard mean difference.

**eTable3 Multivariate Analysis for preoperative DVT in the validation set**

| Characteristics | Univariate | | | Multivariate | | |
| --- | --- | --- | --- | --- | --- | --- |
|  | OR | 95%CI | P-value | OR | 95%CI | P-value |
| Demographic |  |  |  |  |  |  |
| Age, × years | 1.03 | 1.00-1.05 | 0.04 | 1.02 | 0.99-1.05 | 0.15 |
| Female gender | 1.28 | 0.79-2.07 | 0.32 | <NA> | <NA> | <NA> |
| Smoking | 1.88 | 1.08-3.28 | 0.03 | 2.12 | 1.10-4.09 | 0.03 |
| Alcohol | 1.27 | 0.64-2.53 | 0.50 | <NA> | <NA> | <NA> |
| Comorbidities |  |  |  |  |  |  |
| Hypertension | 1.83 | 1.13-2.96 | 0.02 | 1.70 | 0.97-2.98 | 0.06 |
| Diabetes | 1.81 | 1.10-2.99 | 0.02 | 1.74 | 0.99-3.06 | 0.07 |
| COPD | 0.97 | 0.46-2.04 | 0.94 | <NA> | <NA> | <NA> |
| Cardiovascular disease | 1.25 | 0.77-2.04 | 0.37 | <NA> | <NA> | <NA> |
| Stroke | 1.64 | 1.00-2.67 | 0.05 | 0.84 | 0.46-1.54 | 0.58 |
| Chronic liver disease | 1.24 | 0.41-3.68 | 0.71 | <NA> | <NA> | <NA> |
| History of deep venipuncture | 7.19 | 2.26-22.86 | 0.001 | 3.05 | 0.70-13.17 | 0.14 |
| History of deep VTE | 5.18 | 2.92-9.18 | <0.001 | 4.38 | 2.10-9.14 | <0.001 |
| Neoplasms | 0.33 | 010-1.08 | 0.07 | 0.26 | 0.08-0.91 | 0.03 |
| Operation |  |  |  |  |  |  |
| Fracture type | 0.98 | 0.66-1.48 | 0.94 | <NA> | <NA> | <NA> |
| Admission time | 1.28 | 0.99-1.66 | 0.06 | 1.51 | 1.12-2.03 | 0.01 |
| Bedridden time | 1.09 | 1.04-1.14 | <0.001 | 1.09 | 1.04-1.15 | <0.001 |
| Laboratory findings |  |  |  |  |  |  |
| DAR^*^ | 4.21 | 2.45-7.25 | <0.001 | 4.15 | 2.21-7.79 | <0.001 |

*cutoff=0.24, which was identified by Youden’s index.

**eTable4 Patient Characteristics of the validation Set before and After Propensity Score Matching by the best cutoff value of DAR**

| Characteristics | Before matching | | | After matching | | |
| --- | --- | --- | --- | --- | --- | --- |
|  | DAR＜0.24  (n=545) | DAR ≥ 0.24  (n=81) | SMD | DAR＜0.24  (n=80) | DAR ≥ 0.24  (n=80) | SMD |
| Demographic |  |  |  |  |  |  |
| Age, × years | 76.00 (67.00-83.00) | 76.00 (67.00-83.00) | 0.02 | 78.00 (66.00-82.00) | 76.00 (67.00-83.00) | 0.004 |
| Female gender | 313 (57.43) | 49 (60.49) | 0.06 | 51 (63.75) | 49 (61.25) | 0.05 |
| Smoking | 85 (15.60) | 16 (19.75) | 0.11 | 14 (17.50) | 16 (20.00) | 0.06 |
| Alcohol | 61 (11.19) | 10 (12.35) | 0.04 | 10 (12.50) | 10 (12.50) | <0.001 |
| Comorbidities |  |  |  |  |  |  |
| Hypertension | 270 (49.54) | 52 (64.20) | 0.30 | 49 (61.25) | 51 (63.75) | 0.05 |
| Diabetes | 129 (23.67) | 22 (27.16) | 0.08 | 20 (25.00) | 21 (26.25) | 0.03 |
| COPD | 56 (10.28) | 15 (18.52) | 0.24 | 14 (17.50) | 14 (17.50) | <0.001 |
| Cardiovascular disease | 168 (30.83) | 29 (35.80) | 0.11 | 29 (36.25) | 29 (36.25) | <0.001 |
| Stroke | 148 (27.16) | 26 (32.10) | 0.11 | 30 (37.50) | 25 (31.25) | 0.13 |
| Chronic liver disease | 20 (3.67) | 6 (7.41) | 0.16 | 5 (6.25) | 6 (7.50) | 0.05 |
| History of deep venipuncture | 9 (1.65) | 3 (3.70) | 0.13 | 2 (2.50) | 3 (3.75) | 0.07 |
| History of deep VTE | 52 (9.54) | 13 (16.05) | 0.20 | 12 (15.00) | 12 (15.00) | <0.001 |
| Neoplasms | 56 (10.28) | 4 (4.94) | 0.20 | 4 (5.00) | 4 (5.00) | <0.001 |
| Operation |  |  |  |  |  |  |
| Fracture type |  |  | 0.06 |  |  | 0.10 |
| Femoral neck fracture | 251 (46.06) | 35 (43.21) |  | 39 (48.75) | 35 (43.75) |  |
| Intertrochanteric fracture | 270 (49.54) | 42 (51.58) |  | 38 (47.50) | 41 (51.25) |  |
| Subtrochanteric fracture | 24 (4.40) | 4 (4.94) |  | 3 (3.75) | 4 (5.00) |  |
| Admission time |  |  | 0.41 |  |  | 0.07 |
| < 6 h | 275 (50.46) | 56 (69.14) |  | 58 (72.50) | 55 (68.75) |  |
| 6h-12 h | 92 (16.88) | 11 (13.58) |  | 9 (11.25) | 11 (13.75) |  |
| >12 h | 178 (32.66) | 14 (17.28) |  | 13 (16.25) | 14 (17.50) |  |
| Bedridden time | 5.00 (4.00-7.00) | 5.00 (4.00-8.00) | 0.16 | 5.00 (4.00-9.00) | 5.00 (4.00-8.00) | 0.02 |

Data are presented as median (interquartile range) and n (%); DAR: D-dimer – albumin ratio; SMD: standard mean difference.
